# Oral JAK Inhibitors as a Promising Therapeutic Strategy for Refractory Rosacea: A Systematic Review and Meta‐Analysis

**DOI:** 10.1111/jocd.70791

**Published:** 2026-03-08

**Authors:** Yu Peng, Mingzi Sun, Jiayu Chen, Xubo Huang, Fengchuan Zhang

**Affiliations:** ^1^ The Second Clinical Medical College Beijing University of Chinese Medicine Beijing China; ^2^ Department of Dermatology, Dongfang Hospital Beijing University of Chinese Medicine Beijing China

**Keywords:** Abrocitinib, JAK inhibitors, meta‐analysis, rosacea, Tofacitinib, Upadacitinib

## Abstract

**Background:**

Rosacea is a chronic inflammatory dermatosis. While standard therapies exist, managing refractory subtypes remains a therapeutic challenge. Oral Janus kinase (JAK) inhibitors have emerged as potential alternatives, but systematic evidence is lacking.

**Aims:**

To evaluate the efficacy and safety of oral JAK inhibitors for rosacea through a systematic review and meta‐analysis.

**Methods:**

A comprehensive search was conducted in five databases from inception to December 1, 2025. The primary outcome was the pooled clinical response rate. Secondary outcomes included the Investigator's Global Assessment (IGA), Clinician's Erythema Assessment (CEA), and Dermatology Life Quality Index (DLQI).

**Results:**

Eleven studies involving 57 patients were included. The overall pooled response rate was 89.9% (95% CI: 74.4%–99.4%), with low heterogeneity (*I* ^2^ = 16.3%). Regarding secondary outcomes, 67.8% of patients achieved an IGA score of ≤ 1, and 74.6% achieved a CEA reduction of ≥ 2 points. DLQI score also decreased from 17.30 ± 3.80 to 10.00 ± 3.43. Adverse events were reported in 10.5% of patients.

**Conclusions:**

Oral JAK inhibitors show promising potential as a therapeutic option for refractory rosacea. However, given the small sample size and single‐arm design, these findings are exploratory. Large‐scale randomized controlled trials are urgently needed to validate these findings and guide stratified treatment.

## 1 Introduction

Rosacea is a chronic, relapsing inflammatory dermatosis that predominantly affects the central face. It is clinically characterized by a spectrum of signs including transient flushing, persistent erythema, telangiectasia, and inflammatory papules or pustules [1]. With a global prevalence estimated at 5.46% in the adult population, rosacea imposes a substantial disease burden that is often disproportionate to its clinical severity [2, 3]. Due to its visible nature and symptom chronicity, the prevalence rates of anxiety and depression among rosacea patients were reported to be as high as 53.9% and 58.1%, respectively [4]. Current management guidelines recommend topical agents (e.g., ivermectin, metronidazole, brimonidine) and oral anti‐inflammatory antibiotics (e.g., doxycycline) as first‐line therapies [5]. While a significant proportion of patients exhibit inadequate responses or experience frequent relapses upon discontinuation, selecting appropriate systemic therapies for refractory cases remains challenging.

In recent years, Janus kinase (JAK) inhibitors have emerged as a transformative class of drugs in dermatology, demonstrating efficacy in atopic dermatitis, alopecia areata, and vitiligo [6]. Mechanistically, the pathogenesis of rosacea involves the upregulation of cytokines, including interleukin‐17 (IL‐17) and interferon‐γ (IFN‐γ), and the activation of the Toll‐like receptor 2 (TLR2)–kallikrein 5 (KLK5)–cathelicidin (LL‐37) axis, signaling pathways that are closely related to the Janus kinase–signal transducer and activator of transcription (JAK–STAT) cascade [7, 8, 9]. JAK inhibition has been found to downregulate LL‐37‐induced inflammatory cascades and angiogenesis in rosacea models [10]. Although scattered case series suggest rapid symptom control in refractory patients [10, 11, 12, 13, 14, 15, 16, 17, 18, 19], evidence remains limited to scattered, small‐sample observational studies without high‐level synthesis. Given the increasing off‐label use of these agents, a quantitative synthesis of existing data is urgently needed to provide preliminary insights for clinical management and future trial design. Therefore, we conducted the first systematic review and single‐arm meta‐analysis to quantitatively evaluate the efficacy and safety of oral JAK inhibitors for rosacea.

## 2 Materials and Methods

### 2.1 Data Sources and Search Strategy

A comprehensive literature search was conducted in PubMed, Embase, Web of Science, The Cochrane Library, and CNKI (China National Knowledge Infrastructure) from inception to December 1, 2025. The search strategy combined Medical Subject Headings (MeSH) and free‐text terms relevant to “Rosacea” (“acne rosacea”, “Rhinophyma”, “Steroid‐induced rosacea”, “Rosacea‐like dermatitis”) and “JAK inhibitors” (“Janus kinase inhibitors”, “JAKi”, “JAK–STAT”, “Tofacitinib”, “Ruxolitinib”, “Baricitinib”, “Upadacitinib”, “Abrocitinib”). No language restrictions were applied. Our research followed the Preferred Reporting Items for Systematic Reviews and Meta‐Analyses (PRISMA) guidelines [20] (see Supporting Information File S1) and was registered with PROSPERO (CRD420251245235).

### 2.2 Inclusion and Exclusion Criteria

Studies were included if they met the following criteria: (1) Participants: Patients diagnosed with rosacea; (2) Intervention: Treatment with oral JAK inhibitors; (3) Outcomes: Reported clinical efficacy or safety data; (4) Study Design: Randomized controlled trials (RCTs), cohort studies, case series, and case reports.

Studies were excluded if they were: (1) Reviews, meta‐analyses, or animal studies; (2) Studies with insufficient data to extract outcomes; (3) Combined therapies in which the effect of JAK inhibitors could not be isolated.

### 2.3 Data Extraction

Two reviewers independently screened titles, abstracts, and full texts. The following data were extracted using a standardized form: first author, year of publication, study design, sample size, patient characteristics (age, sex, disease duration, prior treatments), rosacea subtype, drug details (name, dosage), treatment duration, and outcomes (clinical response, adverse events). Studies reporting outcomes for distinct patient cohorts (e.g., different rosacea subtypes or treatment arms) within the same publication were treated as independent datasets. Disagreements were resolved through discussion.

### 2.4 Quality and Certainty of Evidence Assessment

The methodological quality of included studies was assessed using the Joanna Briggs Institute (JBI) Critical Appraisal Checklist for Case Report and Case Series. Studies scoring ≥ 70% of items were considered high quality, 50%–69% moderate quality, and < 50% low quality. The certainty of evidence was assessed using the GRADE (Grading of Recommendations Assessment, Development and Evaluation) approach. Assessments were performed by two independent reviewers. Disagreements were resolved by discussion.

### 2.5 Statistical Analysis

All statistical analyses were performed using Stata 18.0 software. For individual studies, response rates and 95% confidence intervals (CIs) were calculated using the Clopper‐Pearson exact method. Given that the included studies were primarily case series with small sample sizes and several studies reported extreme response rates (100%), we utilized the Freeman‐Tukey double arcsine transformation. A random‐effects model (DerSimonian‐Laird estimator) was utilized to pool the effect size. Heterogeneity was assessed using the *I* ^2^ statistic and Cochran's Q test. An *I* ^2^ < 50% was considered to indicate low‐to‐moderate heterogeneity. Subgroup analyses were conducted based on drug type and clinical phenotype. Sensitivity analysis was performed by sequentially removing individual studies to evaluate the robustness of the results. Publication bias was assessed using funnel plots and Egger's linear regression test. A two‐sided *P*‐value < 0.05 was considered statistically significant.

## 3 Results

### 3.1 Study Characteristics

**FIGURE 1:**
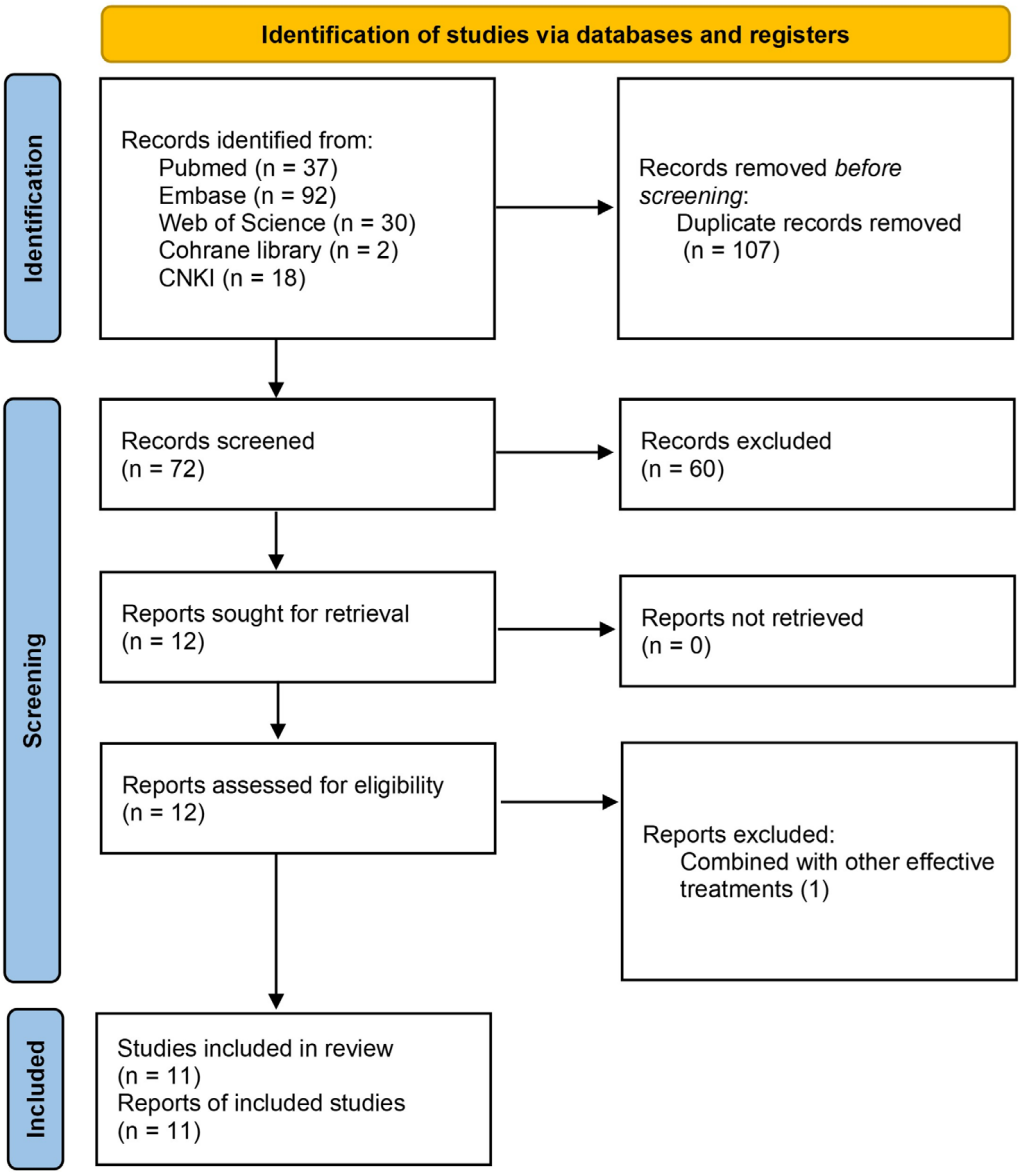
Flow chart.

A total of 11 studies involving 57 patients were included (Figure 1), comprising 52 females and 5 males. The detailed characteristics of these studies are summarized in Table 1. The study population primarily consisted of middle‐aged women, with a mean age of 36.6 ± 9.0 years (range, 22–56 years). The mean disease duration was 2.6 ± 2.5 years, ranging from 1 month to 10 years.

**TABLE 1:** Characteristics of Included Studies.

Study	Design	N	Age (y), Mean ± SD (range)	Female, *n* (%)	Subtype	Disease duration	Intervention	Treatment duration	Prior treatments
Mao 2025	Case report	1	29	1 (100.0%)	IPL‐aggravated	several months	Abrocitinib 200 mg qd (tapered)	12w	Minocycline, topical metronidazole, azelaic acid, IPL, prednisone
Xu 2025	Real world study	12	36.6 ± 10.2 (22–56)	9 (75.0%)	PPR	3.1 ± 2.5 (0.5–8) y	Upadacitinib 15 mg qd	12w	No systemic therapy within 3 months
Meng 2024	Case series	6	32.7 ± 8.4 (23–47)	6 (100.0%)	SIR	1.0 ± 0.8 (0.25–2.5) y	Abrocitinib 100/200 mg qd (tapered)	10‐24w (ongoing)	Minocycline (2), metronidazole (1), carvedilol (1), compound glycyrrhizin (4), methotrexate (3)
Sun 2024	Case series	3	NR	NR	ETR	NR	Tofacitinib	4w	NR
Teng 2024	Case series	3	41.7 ± 11.5 (35–55)	3 (100.0%)	ETR	4.7 ± 4.7 (1–10) y	Abrocitinib 100 mg qd (tapered)	16‐18w	Doxycycline (2), hydroxychloroquine (3), carvedilol (2), IPL (2)
Zhang 2024 (Group A)	Case series	2	36.5 ± 12 (28–45)	2 (100.0%)	ETR	2.5 ± 0.7 (2–3) y	Upadacitinib 15 mg qd	4 (lost to follow‐up)‐12w (ongoing)	All patients received comprehensive therapies: minocycline, hydroxychloroquine, carvedilol, physical therapy etc.
Zhang 2024 (Group B)	Case series	4	37.8 ± 2.5 (35–41)	4 (100.0%)	ETR	3.3 ± 0.5 (3–4) y	Abrocitinib 100 mg qd	12‐20w	
Cao 2023	Case series	3	45.0 ± 4.4 (40–48)	3 (100.0%)	PPR	4.3 ± 3.1 (1–7) y	Tofacitinib 5 mg bid (tapered)	6 m	Doxycycline (3), hydroxychloroquine(3), topical calcineurin inhibitors(2), topical metronidazole (2), topical corticosteroids (1) Oral corticosteroids (2), topical clobetasol(1)
Ren 2023	Case report	1	53	1 (100.0%)	GR	1 m	Abrocitinib 100 mg qd (tapered)	20w	None
Xu 2023	Case series	4	40.0 ± 10.0 (35–55)	4 (100.0%)	SIR	1 – 2y	Abrocitinib 100 mg qd (tapered)	2 (lost to follow‐up)‐ 8w (ongoing)	Hydroxychloroquine (2), macrolide antibiotics (2), betamethasone intramuscularly (2)
Li 2022	Case report	1	28	1 (100.0%)	SIR	2y	Tofacitinib 5 mg bid (tapered)	6w	Hydroxychloroquine, minocycline, topical metronidazole, tacrolimus, crisaborole
Sun 2022 (Group A)	Case series	8	31.3 ± 6.2 (24–41)	6 (75.0%)	PPR	3.2 ± 3.6 (0.25–10)	Tofacitinib 5 mg bid (tapered)	2–10 w	Minocycline (2), doxycycline (3), hydroxychloroquine (1), metronidazole (2), ivermectin (1), clarithromycin (1), tretinoin (1),
Sun 2022 (Group B)	Case series	9	37.6 ± 9.3 (24–49)	9 (100.0%)	ETR	1.3 ± 1.3 (0.25–4)	Tofacitinib 5 mg bid (tapered)	4‐8w	Minocycline (1), doxycycline (2), hydroxychloroquine (4), topical calcineurin inhibitors (1), carvedilol (1), isotretinoin (2), antihistamine (1)

Abbreviations: ETR, erythematous telangiectatic rosacea; GR, granulomatous rosacea; IPL, intense pulsed light; NR, not reported; PPR, papulopustular rosacea; SD, standard deviation; SIR, steroid‐induced rosacea.

In terms of clinical subtypes, there were 23 patients with papulopustular rosacea (PPR) and 21 with erythematotelangiectatic rosacea (ETR). Thirteen patients presented with special refractory subtypes characterized by intense inflammatory reactions, including 11 cases of steroid‐induced rosacea (SIR), 1 case of granulomatous rosacea (GR), and 1 case of intense pulsed light (IPL)‐aggravated rosacea. The majority of patients had a history of inadequate response to conventional therapies. Commonly reported prior treatments included oral medications (e.g., minocycline, hydroxychloroquine, carvedilol), topical agents (e.g., metronidazole, calcineurin inhibitors), and physical therapies.

Patients were treated with oral JAK inhibitors, specifically tofacitinib, abrocitinib, and upadacitinib. The dosing regimens varied across studies. Tofacitinib was typically administered at 5 mg twice daily, upadacitinib at 15 mg once daily, and abrocitinib dosages ranged from 100 mg to 200 mg daily, often with dose tapering based on clinical response. Efficacy was evaluated primarily based on clinical response rates, while standardized metrics including IGA, CEA, and DLQI were reported in some studies.

### 3.2 Primary Outcome

**FIGURE 2:**
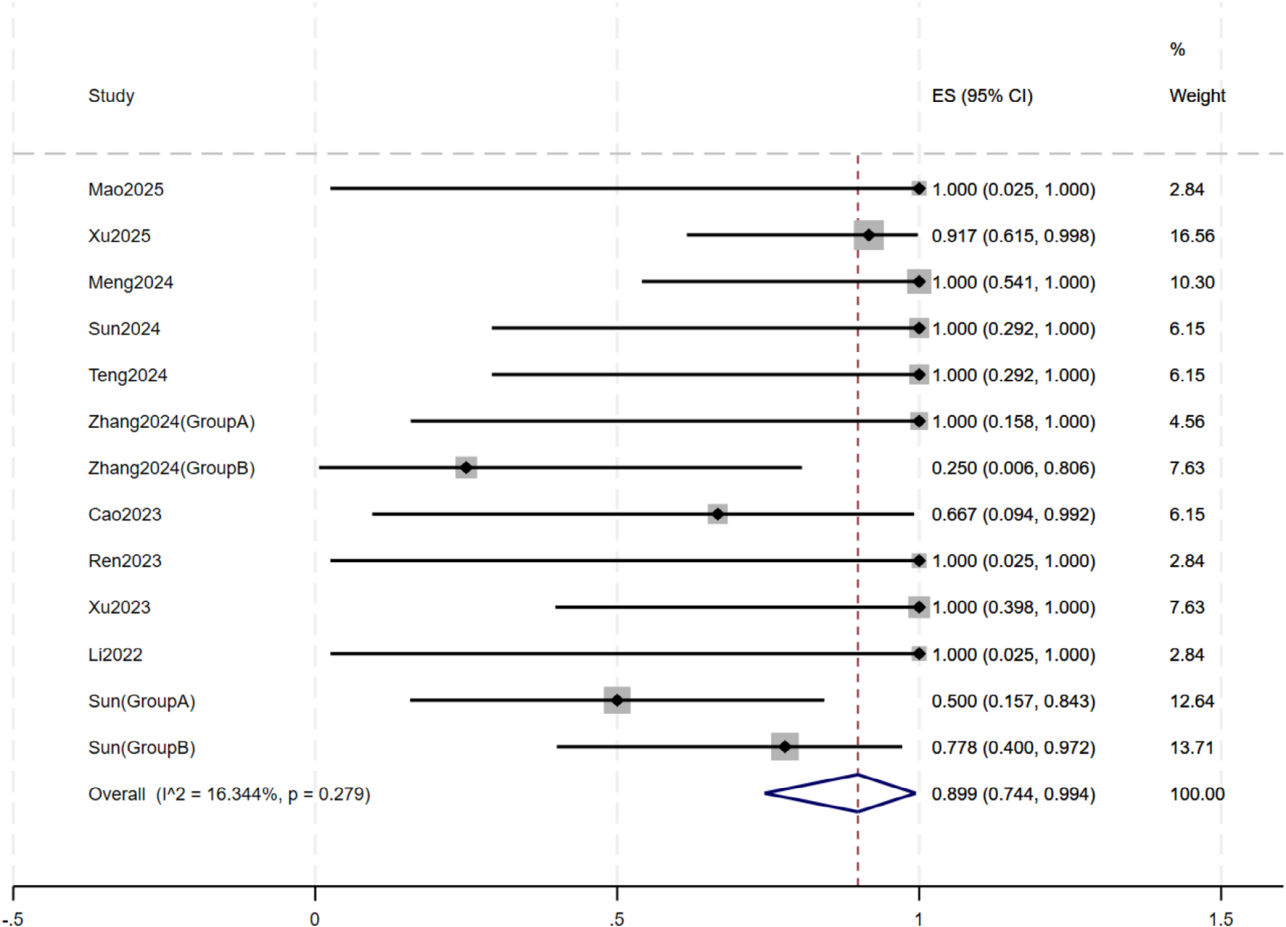
Forest plot of overall efficacy.

A total of 13 study subgroups were included in the primary efficacy analysis. The overall pooled response rate of oral JAK inhibitors for rosacea was 89.9% (95% CI: 74.4%–99.4%) (Figure 2). The individual response rates across studies ranged from 25.0% to 100.0%. The overall heterogeneity was low (*I* ^2^ = 16.3%, *p* = 0.28).

### 3.3 Subgroup Analyses

Subgroup analyses were performed based on disease subtype and drug type.

#### 3.3.1 Disease Subtype (Figure 3)

**FIGURE 3:**
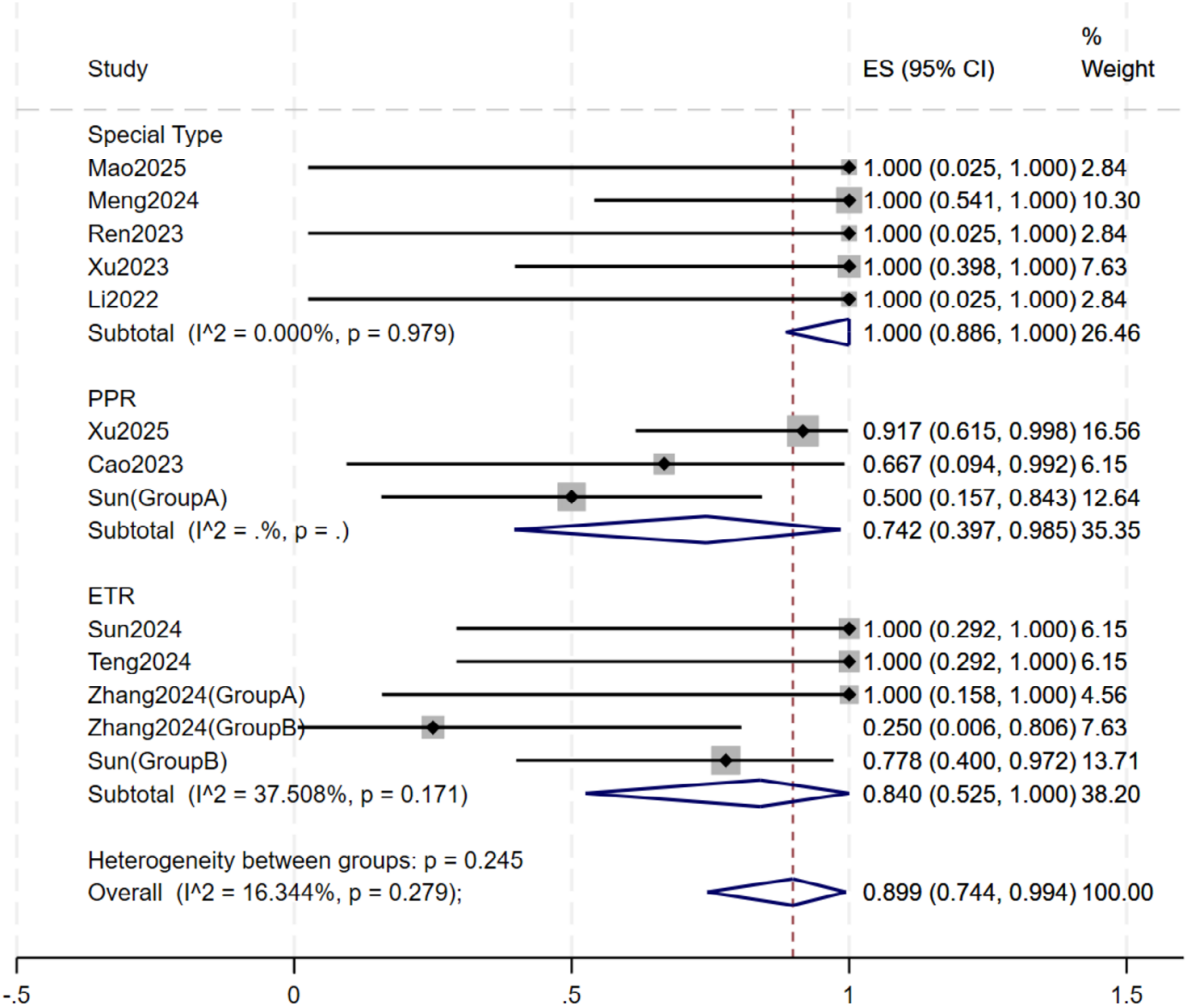
Forest plot of subgroup by disease subtype.

Patients were categorized into three groups based on rosacea subtype: PPR, ETR, and Special Types. Oral JAK inhibitors suggested the highest numerical response rate in the Special Types group, with a response rate of 100% (95% CI:88.6% – 100.0%), followed by the ETR group (84.0%, CI: 52.5% –100%) and the PPR group (74.2%, 95% CI: 39.7% – 98.5%). No statistically significant difference was observed between the subgroups (*p* = 0.25). In the PPR subgroup, the study utilizing upadacitinib reported a high response rate of 91.7% (11/12), and the two studies employing tofacitinib yielded a lower pooled response rate of 54.5% (6/11). No studies reporting the use of abrocitinib for PPR were identified. In the ETR subgroup, only two patients were treated with upadacitinib, and both achieved a clinical response (2/2). The pooled response rates of tofacitinib and abrocitinib for ETR were 83.3% (10/12) and 57.1% (4/7), respectively. The response rates of ETR ranged from 25.0% to 100.0%. In the Special Type subgroup, all patients achieved clinical response following treatment with oral JAK inhibitors.

#### 3.3.2 Drug Subtype (Figure 4)

**FIGURE 4:**
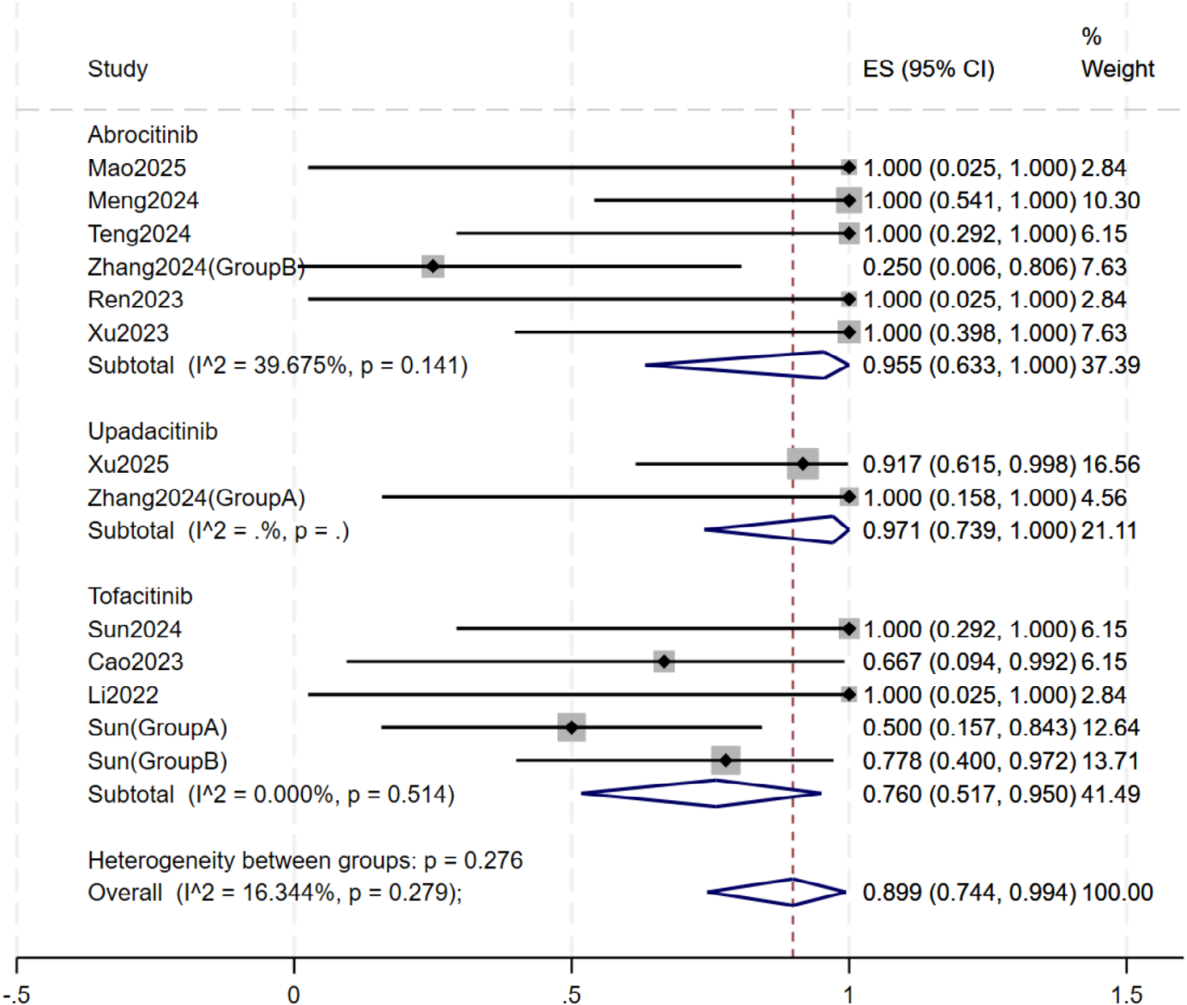
Forest plot of subgroup by drug type.

Patients were stratified into three groups based on the oral JAK inhibitor administered: abrocitinib, tofacitinib, and upadacitinib. The upadacitinib group exhibited the highest response rate of 97.1% (95% CI: 73.9% – 100%), followed by the abrocitinib group at 95.5% (95% CI: 63.3% – 100%) and the tofacitinib group at 76.0% (95% CI: 51.7% – 95.0%). The difference between drug subgroups was not statistically significant (*p* = 0.28).

### 3.4 Secondary Outcomes

#### 3.4.1 Clinical Symptoms

**FIGURE 5:**
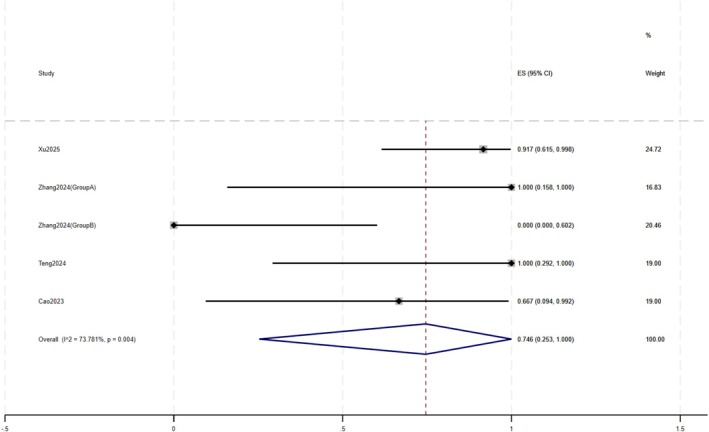
Forest plot of CEA reduction ≥ 2.

**FIGURE 6:**
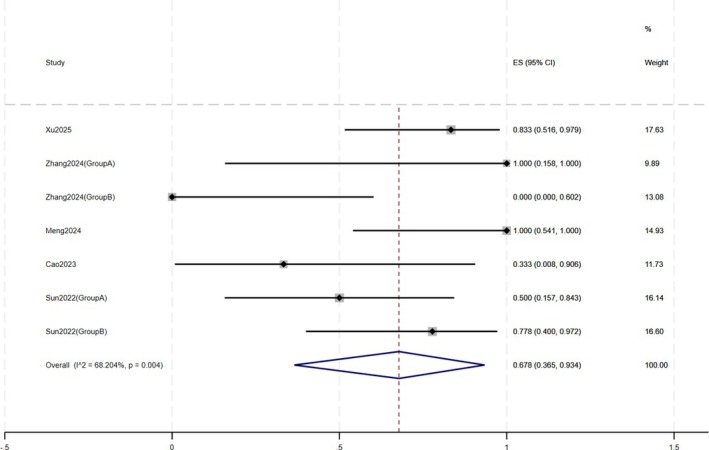
Forest plot of IGA ≤ 1.

Secondary outcomes also indicated clinical improvements. Some studies reported changes in CEA, IGA, and DLQI scores in patients before and after treatment. Regarding CEA (Figure 5), 74.6% (95% CI: 25.3%–100%) of patients achieved a reduction of ≥ 2 points, with the mean CEA score in 12 patients decreasing from 2.92 ± 0.79 to 1.33 ± 0.89. After treatment, 67.8% (95% CI: 36.5%–93.4%) of patients achieved an IGA score of ≤ 1. In 29 patients, the mean IGA score decreased from 3.41 ± 0.57 to 1.52 ± 0.78 (Figure 6). The mean DLQI score in 10 patients decreased from 17.30 ± 3.80 to 10.00 ± 3.43.

#### 3.4.2 Time to Response and Reduction

Rapid improvement was a common feature. Most studies reported visible reduction in erythema or pruritus within 1 to 2 weeks. Zhang et al. observed symptom relief as early as 48 h after initiating upadacitinib [14]. Doses were tapered based on response between weeks 2 and 20, mostly within 2–4 weeks for patients with favorable outcomes. The regimen involved halving the dose or alternate‐day dosing, then decreasing to twice weekly until discontinuation. One patient experienced a transient rebound of erythema, pruritus, and flushing following tapering [14]. After drug withdrawal, 25 patients in 5 studies were followed up, with the follow‐up period ranging from 2 weeks to 6 months [11, 12, 17, 18, 19]. Among them, 7 patients treated with tofacitinib relapsed [19], while the other patients did not relapse.

#### 3.4.3 Safety Evaluation

The overall incidence of adverse events (AEs) was 10.5% (6/57). A total of 6 patients experienced AEs during treatment, 3 of whom discontinued therapy. Five patients reported digestive system‐related AEs. Specifically, two patients presented with gastrointestinal symptoms (e.g., anorexia, nausea, vomiting) [12, 21], of these, one continued treatment, while the other successfully restarted therapy after an 8‐week suspension without recurrence of these symptoms. Regarding hepatic abnormalities, one patient discontinued treatment due to HBV DNA positivity with elevated transaminases [14], and another withdrew due to elevated liver enzymes [19]. A third patient presented with elevated bilirubin, which was managed by dose reduction [19]. Additionally, one patient discontinued therapy due to worsening facial erythema accompanied by mild eosinophilia and basophilia [15].

### 3.5 Sensitivity Analysis and Publication Bias

Sensitivity analysis using the leave‐one‐out method confirmed the robustness of our findings (Supplementary Figure S1). No single study disproportionately influenced the overall outcome. Funnel plot (Supplementary Figure S2) showed a relatively symmetrical distribution of studies. Egger's test yielded a *P*‐value of 0.67, providing no evidence of significant publication bias.

### 3.6 Quality and Evidence Assessment

Quality assessment using the JBI checklist indicated that 7 studies were classified as high quality, and the remaining 4 studies were considered moderate quality. No studies were categorized as low quality. The primary limitation was the unclear reporting of consecutive inclusion, as most studies failed to specify enrollment methods. Complete inclusion and clinic demographics were also frequently unreported. Detailed results are provided in Supplementary Table S1.

The certainty of evidence for all outcomes was rated as ‘Very Low’ according to GRADE. Primary limitations included the observational design and serious imprecision. We also downgraded for potential publication bias inherent to case series and case reports, despite the symmetrical funnel plots. Additionally, inconsistency affected CEA and IGA outcomes (see Supplementary Table S2).

## 4 Discussion

To the best of our knowledge, this is the first single‐arm meta‐analysis to evaluate the use of oral JAK inhibitors for rosacea. Our study found that oral JAK inhibitors showed a high overall pooled response rate of 89.9% (95% CI: 74.4%–99.4%) in rosacea. Marked clinical improvements were observed. Mean IGA, CEA, and DLQI scores decreased following treatment. 67.8% (95% CI: 36.5%–93.4%) of patients achieved an IGA score of ≤ 1, and 74.6% (95% CI: 25.3%–100%) achieved a CEA reduction of ≥ 2 points. These findings indicate the efficacy of oral JAK inhibitors for rosacea, suggesting they may serve as a promising therapeutic alternative, particularly for patients who have failed conventional therapies or present with severe inflammatory reactions.

The efficacy of JAK inhibitors is intrinsically linked to the underlying pathogenesis of rosacea. Innate immunity dysregulation is a primary pathogenic driver, with the TLR2‐KLK5‐LL37 axis serving as a critical pathway [9]. TLR2 is overexpressed in keratinocytes of rosacea patients, thereby heightening cutaneous inflammatory responses to environmental stimuli [22]. Triggers such as *Demodex* mites or ultraviolet radiation activate TLR2, which in turn induces the release of KLK5, and KLK5 subsequently cleaves the precursor cathelicidin into its active proinflammatory peptide fragment, LL‐37 [23]. As a central mediator in rosacea pathogenesis, LL‐37 not only triggers a cascade of inflammatory reactions but also induces angiogenesis and telangiectasia [23, 24]. Furthermore, LL‐37 can bind to TLR2, initiating downstream inflammatory cascades and perpetuating a vicious cycle [25]. The JAK–STAT pathway functions as one of the key downstream signaling pathways of the TLR2‐KLK5‐LL37 axis [9]. JAK inhibitors exert their therapeutic effects by blocking this critical signaling pathway, thereby suppressing the release of downstream proinflammatory cytokines and alleviating clinical symptoms. Additionally, JAK inhibitors may contribute to therapeutic outcomes by improving skin barrier function, modulating neurovascular regulation, mitigating oxidative stress, and alleviating pruritus [26].

Our study found that JAK inhibitors showed a high response rate of 100% in patients with Special Types. This subgroup, characterized by intense inflammation, comprised SIR, granulomatous rosacea, and IPL‐aggravated rosacea. In one case of laser‐aggravated rosacea, facial erythema remained refractory to 60 mg prednisone but was rapidly controlled following the initiation of abrocitinib. This suggests the ability of JAK inhibitors to rapidly arrest inflammatory responses. Taking SIR as a prime example of special types, glucocorticoids are known to upregulate TLR2 expression in the skin [27]. Long‐term topical use suppresses cutaneous immune responses; however, upon withdrawal, an immune rebound occurs, characterized by a massive release of cytokines such as IL‐6, IL‐10, and IFN‐γ [17]. Since the signaling of these cytokines relies on JAK–STAT pathways, both highly selective JAK1 inhibitors and pan‐JAK inhibitors can rapidly and potently block their downstream signaling, effectively controlling acute inflammation. In contrast, the response rates for ETR (84.0%) and PPR (74.2%) were numerically lower than that of the special types, though this difference did not reach statistical significance. While the pathogenesis in special subtypes is dominated by intense inflammatory reactions, ETR and PPR are also intimately associated with neurovascular dysregulation and microbiome disturbances. Given that JAK inhibitors primarily function by suppressing inflammation, their therapeutic efficacy in addressing these non‐inflammatory components may be limited.

In the ETR subgroup, upadacitinib showed a 100% response (2/2), followed by tofacitinib at 83.3% (10/12) and abrocitinib at 57.1% (4/7). These drug‐specific differences are strictly constrained by the small sample sizes, particularly for the upadacitinib subgroup, in which only two patients were included. However, we observed variability in efficacy across individual studies, with response rates ranging from 25.0% to 100.0%. This difference likely reflects the complex pathogenesis of ETR, which is clinically characterized by persistent central facial erythema, paroxysmal flushing, and telangiectasia. Flushing in ETR is predominantly driven by neurovascular dysregulation, and Transient Receptor Potential (TRP) ion channels—specifically TRPV1–4 and TRPA1—play a major role [28]. They mediate sensory and inflammatory signaling, and trigger burning sensations and vasodilation [28, 29]. Although certain JAK inhibitors, such as tofacitinib, have been shown to inhibit TRPV1 [30], their impact on other critical nodes of the TRPV/TRPA pathway remains ill‐defined. Therefore, the therapeutic effect of JAK inhibitors on neurovascular‐mediated paroxysmal flushing may be limited. Regarding telangiectasia, JAK inhibitors can modulate angiogenesis via the JAK/STAT3 signaling pathway, which regulates angiogenesis by modulating the numbers of angiogenesis‐related growth factors and regulating the cell cycle [31]. However, for established, long‐standing telangiectasia characterized by fixed structural changes, the efficacy may be limited. Long‐term use of JAK inhibitors may help prevent structural progression by preventing neovascularization and reducing erythema associated with perivascular inflammation. In conclusion, the variability in ETR efficacy likely correlates with specific lesion characteristics. JAK inhibitors appear highly effective for patients with predominant inflammation‐mediated persistent erythema, whereas their efficacy may be limited for those dominated by paroxysmal flushing or fixed telangiectasia.

In the PPR subgroup, upadacitinib achieved a high response rate of 91.7% (11/12), whereas tofacitinib showed a lower rate of 54.5% (6/11). The efficacy of tofacitinib for PPR is also lower than that for ETR (83.3%, 10/12). This discrepancy may be attributed to the pharmacological profile of tofacitinib as a pan‐JAK inhibitor and the unique pathophysiology of PPR. PPR is characterized by a significantly higher density of *Demodex* mite compared to ETR [32]. Mechanistically, tofacitinib inhibits JAK3, which mediates the signaling of the common γ‐chain cytokine family (e.g., IL‐15, IL‐7) [8]. These cytokines are essential for the maintenance of lymphocyte homeostasis and natural killer cell function. Compared to high selective JAK1 Inhibitors, broad JAK3 inhibition may compromise the host's innate defense against the high *Demodex* burden. Clinical evidence supports this hypothesis. A previous case reported severe *Demodex* folliculitis in a patient treated with tofacitinib for ulcerative colitis [33]. Similarly, one PPR patient in our review discontinued tofacitinib due to expanded lesion area and increased papule and pustule counts [15]. Therefore, we postulate that in PPR, tofacitinib‐induced local immune suppression may lead to unchecked *Demodex* proliferation. The increased microbial load continuously activates TLR2, generating new inflammatory signals. These signals offset the drug's anti‐inflammatory effects, hindering clearance or even exacerbating the disease. In contrast, highly selective JAK1 inhibitors have minimal impact on JAK3 activity. While effectively blocking pathogenic cytokines, they relatively preserve antimicrobial immune surveillance. By controlling inflammation without compromising host defense against *Demodex*, selective JAK1 inhibitors may represent a theoretically superior therapeutic option for PPR.

Beyond mechanistic insights, our findings offer a theoretical basis for future clinical research. Current evidence suggests positioning JAK inhibitors primarily as a promising alternative strategy for refractory rosacea. They appear most suitable for patients who have failed standard treatment, or for those with steroid‐induced rosacea requiring rapid stabilization. Theoretically, selective JAK1 inhibitors might be preferable given the high Demodex burden for PPR. For SIR or ETR, tofacitinib remains a potential and more affordable option, as these forms are driven less by microbial load. These hypotheses require validation in head‐to‐head randomized controlled trials before guiding clinical selection. In terms of dosing, the most common effective regimens were tofacitinib 5 mg twice daily, or upadacitinib 15 mg and abrocitinib 100 mg once daily. Rapid onset is a key feature, often visible within 2 weeks. Consequently, a 4‐week trial period could be considered as a potential decision point. If no clear improvement is observed by then, the continuation of therapy should be critically re‐evaluated. Abrupt cessation should be avoided to minimize cytokine‐rebound risks. Successful protocols typically involve a gradual tapering phase over 2 to 4 weeks once remission is achieved. Skin lesions remained effectively controlled in the vast majority of patients during the tapering phase, with only a single reported case of transient symptom rebound.

Overall, oral JAK inhibitors exhibited an acceptable safety profile within the short follow‐up period. The majority of adverse events are mild and transient gastrointestinal symptoms. Given the limited follow‐up, strict vigilance is required regarding HBV reactivation and JAK inhibitor‐associated acne. The observation of HBV DNA reactivation in one patient warrants particular attention. This underscores the necessity of rigorous pre‐treatment screening for contraindications and regular laboratory monitoring throughout the treatment course. Additionally, clinicians should remain vigilant regarding the potential risk of JAK inhibitor‐associated acne. Accumulating evidence suggests that the use of JAK inhibitors in other dermatological diseases is associated with an elevated risk of acne [34, 35]. Therefore, when treating rosacea patients with JAK inhibitors, clinicians should carefully distinguish between a rosacea flare and JAK‐induced acne. The former is characterized by intensifying erythema and vasomotor instability, whereas the latter is driven by follicular occlusion and comedogenesis. However, data on long‐term outcomes and recurrence rates after discontinuation are currently lacking. Vigilance is mandatory regarding potential adverse events that may arise over longer periods.

Our study has several limitations that should be acknowledged. The primary constraint lies in the study design, as all included studies were observational designs lacking control groups. Therefore, we cannot rule out placebo effects or the natural fluctuation of the disease. Unlike prospective designs with standardized data collection and pre‐defined endpoints, our retrospective data is inherently subject to inconsistencies in reporting and patient selection bias. Given the ‘Very Low’ certainty of the current evidence, verification through high‐quality randomized designs is indispensable. Our search of the Cochrane Library identified 2 ongoing clinical trials (ChiCTR2400093737, ChiCTR2500109214) investigating the efficacy and safety of oral JAK inhibitors for rosacea. The completion of these trials will be pivotal in validating our findings and establishing standardized treatment protocols. Additionally, although we incorporated the most recent evidence, the total sample size (*N* = 57) remains relatively small, which limits the statistical power and robustness of our estimates. Thus, the results should be viewed as hypothesis‐generating rather than definitive. Geographical generalizability is another concern, as the cohort was exclusively Asian (Chinese), potentially limiting generalizability to White populations with different genetic backgrounds. Finally, most studies had short follow‐up periods, thus data on long‐term safety and recurrence rates after discontinuation are currently lacking.

## 5 Conclusion

In conclusion, preliminary evidence suggests that oral JAK inhibitors offer a potential therapeutic alternative for recalcitrant rosacea, particularly for patients failing conventional regimens. While statistical significance was not reached, based on the observed numerical trends and mechanistic rationale, we raise the hypothesis that a stratified approach might warrant investigation in future trials. Although severe adverse events were rare, the potential for systemic risks necessitates rigorous pre‐treatment screening and continuous monitoring. Future research should prioritize large‐scale randomized controlled trials to establish long‐term safety profiles and identify the optimal JAK inhibitor tailored to specific clinical subtypes.

## Author Contributions

F.Z. conceived and designed the study. Y.P. and M.S. conducted the literature search, screened the studies, and extracted the data independently. Any discrepancies were resolved through discussion with J.C. and X.H., Y.P. performed the statistical analysis and drafted the manuscript. J.C. and X.H. assisted in the visualization and critically revised the manuscript. F.Z. provided supervision and final approval of the version to be published.

## Funding

This work was supported by National High Level Chinese Medicine Hospital Clinical Research Funding.

## Disclosure

Declaration of Generative AI and AI‐assisted technologies in the writing process: AI tools (e.g., DeepL) were used to assist with translation and linguistic refinement. The authors reviewed and edited the translated text to ensure accuracy and scientific integrity. All scientific content and analyses were conducted and verified by all authors.

Study Registration: PROSPERO (CRD420251245235).

## Ethics Statement

The authors have nothing to report.

## Conflicts of Interest

The authors declare no conflicts of interest.

## Supporting information


**File S1:** PRISMA 2020 checklist.

**Table S1:** Quality assessment.

**Table S2:** GRADE assessment.

**Figure S1:** Sensitivity analysis.

**Figure S2:** Funnel plot.

## Data Availability

The data presented in this study are available on request from the corresponding author.
