# Supplementary figures and images for: Oral JAK Inhibitors as a Promising Therapeutic Strategy for Refractory Rosacea: A Systematic Review and Meta‐Analysis
Source: J Cosmet Dermatol. 2026 Mar 8;25(3):e70791. doi: 10.1111/jocd.70791 (PMC12968368; doi:10.1111/jocd.70791)

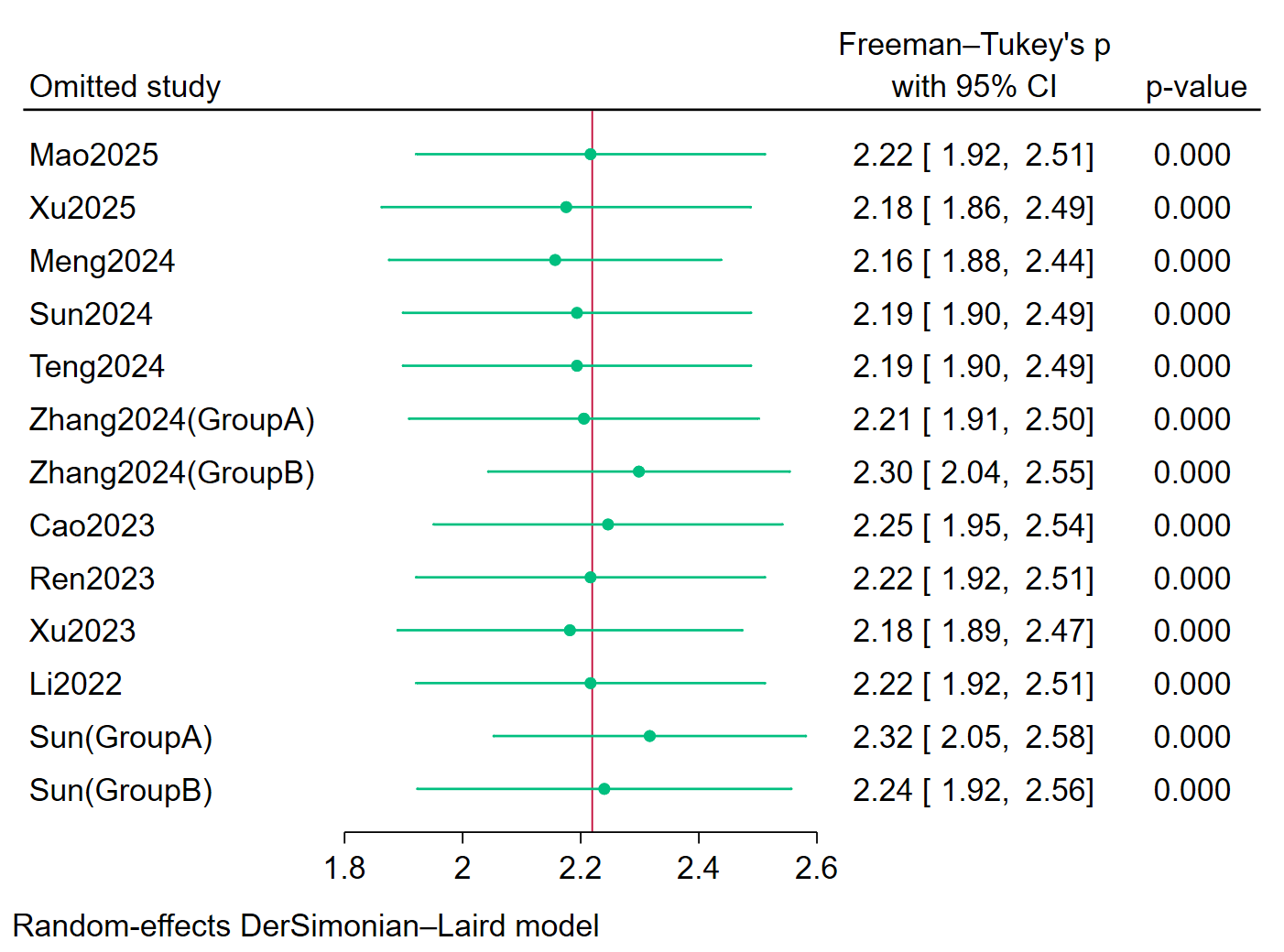

Supplement: Supplementary file 4 — Figure S1: Sensitivity analysis. [file JOCD-25-e70791-s002.png]

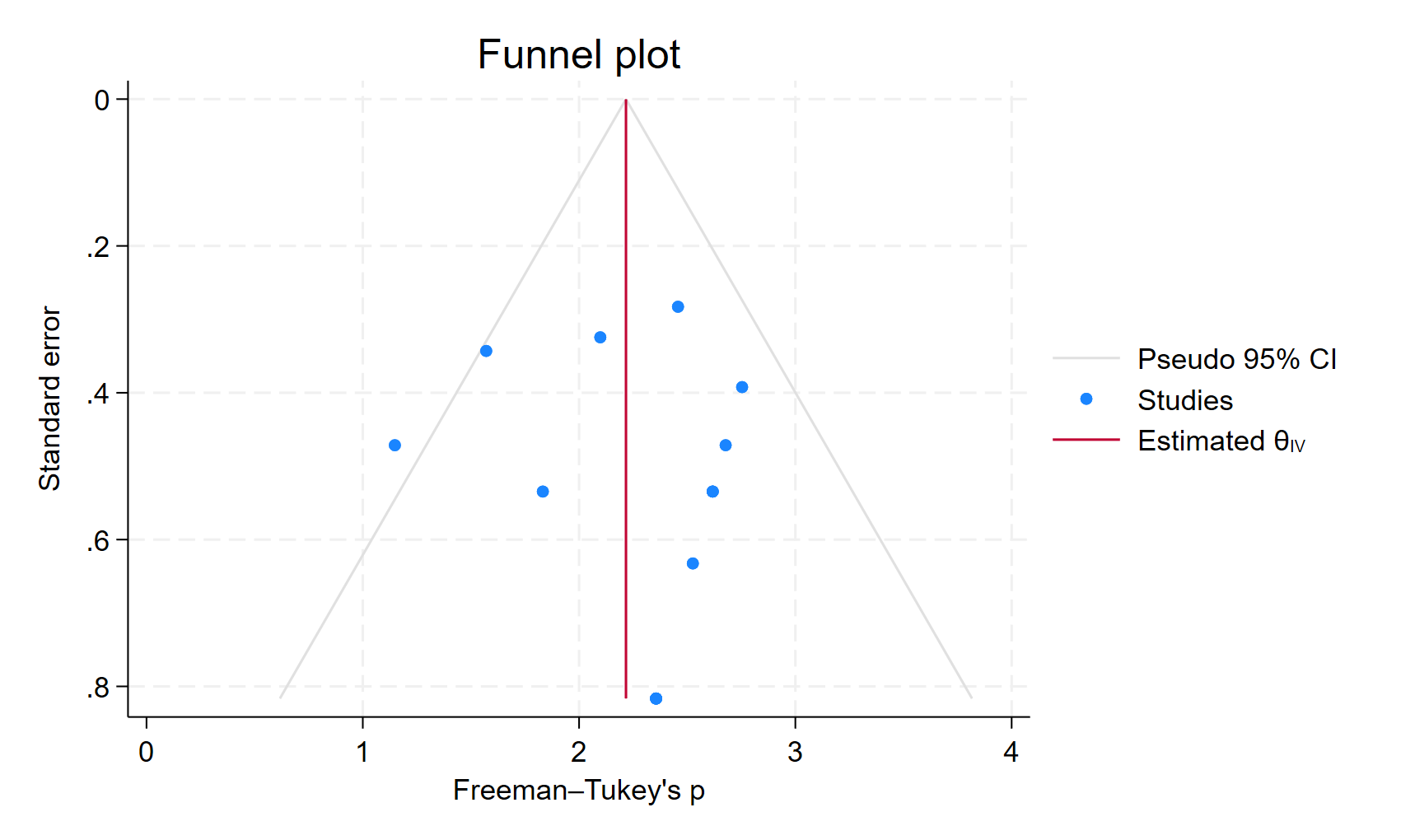

Supplement: Supplementary file 5 — Figure S2: Funnel plot. [file JOCD-25-e70791-s001.png]
